# Out of the bush: the Asian bush mosquito *Aedes japonicus japonicus* (Theobald, 1901) (Diptera, Culicidae) becomes invasive

**DOI:** 10.1186/1756-3305-7-59

**Published:** 2014-02-04

**Authors:** Helge Kampen, Doreen Werner

**Affiliations:** 1 Friedrich-Loeffler-Institut, Federal Research Institute for Animal Health, Südufer 10, Greifswald – Insel Riems 17493, Germany; 2 Leibniz Centre for Agricultural Landscape Research, Eberswalder Str. 84, Müncheberg 15374, Germany

**Keywords:** *Aedes japonicus japonicus*, Asian bush mosquito, Asian rock pool mosquito, Europe, International spread, Invasive species, North America, Vector potential

## Abstract

The Asian bush or rock pool mosquito *Aedes japonicus japonicus* is one of the most expansive culicid species of the world. Being native to East Asia, this species was detected out of its original distribution range for the first time in the early 1990s in New Zealand where it could not establish, though. In 1998, established populations were reported from the eastern US, most likely as a result of introductions several years earlier. After a massive spread the mosquito is now widely distributed in eastern North America including Canada and two US states on the western coast. In the year 2000, it was demonstrated for the first time in Europe, continental France, but could be eliminated. A population that had appeared in Belgium in 2002 was not controlled until 2012 as it did not propagate. In 2008, immature developmental stages were discovered in a large area in northern Switzerland and bordering parts of Germany. Subsequent studies in Germany showed a wide distribution and several populations of the mosquito in various federal states. Also in 2011, the species was found in southeastern Austria (Styria) and neighbouring Slovenia. In 2013, a population was detected in the Central Netherlands, specimens were collected in southern Alsace, France, and the complete northeastern part of Slovenia was found colonized, with specimens also present across borders in adjacent Croatia. Apparently, at the end of 2013 a total of six populations occurred in Europe although it is not clear whether all of them are completely isolated. Similarly, it is not known whether these populations go back to the same number of introductions. While entry ports and long-distance continental migration routes are also obscure, it is likely that the international used tyre trade is the most important mode of intercontinental transportation of the mosquito. *Aedes j. japonicus* does not only display an aggressive biting behaviour but is suspected to be a vector of various disease agents and to displace indigenous culicid species. Therefore, *Aedes j. japonicus* might both cause public health problems in the future and have a significant impact on the biodiversity of the invaded territories.

## Introduction

Among vector arthropods invading (i.e. arriving, establishing and spreading in) new territories, mosquitoes have historically been especially successful [1]. A highly ranked listed invasive culicid species [2] is the Asian bush mosquito or Asian rock pool mosquito *Aedes* (*Finlaya*) *japonicus japonicus* (Theobald, 1901). It originates from East Asia and the Far East, where it represents one of four subspecies of *Ae. japonicus*. Similar to *Aedes albopictus*, it is transported to overseas territories mainly by the used tyre trade. Up to now, it has been discovered in Oceania (New Zealand), where it could not establish thanks to thorough inspection activities and early detection, as well as in North America and Europe, with the first detections outside its native distribution area dating back to the early 1990s (Table 1). We here summarize key data on the international spread, the biology and the harmful potential of *Ae. j. japonicus*.

**Table 1:** **Dates of first detection of invasion/establishment by *Ae. j. japonicus***

**Year of first detection**	**Country/state**	**Established**	**Reference**
1993	**New Zealand**		[3]
	**United States**	**-**	
1998	New Jersey	**+**	[4]
1998	New York	**+**	[4]
1998	Connecticut	**+**	[5]
1999	Pennsylvania	**+**	[6]
1999	Ohio	**+**	[7]
1999	Rhode Island	**+**	[8]*
2000	New Hampshire	**+**	[9]
2000	Massachusetts	**+**	[10]
2000	Maryland	**+**	[11]
2000	Virginia	**+**	[12]
2001	Delaware	**+**	[8]*
2001	Maine	**+**	[13]
2001	Vermont	**+**	[14]
2001	Washington	**+**	[15]
2002	Georgia	**+**	[16,17]
2002	South Carolina	**+**	[16]
2002	West Virginia	**+**	[18]
2003	North Carolina	**+**	[17]
2003	Tennessee	**+**	[19]
2003	Kentucky	**+**	[20]
2003	Hawaii	**+**	[21]
2003	Michigan	**+**	[22]
2004	Alabama	**+**	[23]
2004	Wisconsin	**+**	[24]
2004	Indiana	**+**	[25]
2005	Missouri	**+**	[26]
2006	Illinois	**+**	[27]
2006	Oregon	**+**	[28]
2007	Minnesota	**+**	[29]
2007	Iowa	**+**	[30]
2009	South Dakota	**+**	[31]
2010	Arkansas	**+**	[32]
2011	Mississippi	**+**	[33]
	**Canada**		
2001	Quebec	**+**	[34]
2001	Ontario	**+**	[35]
	**Europe**		
2000	France	**–**	[36]
2002	Belgium	**+**	[37]
2008	Switzerland	**+**	[38]
2008	Germany	**+**	[38]
2011	Austria	**+**	[39]
2011	Slovenia	**+**	[39]
2013	Netherlands	**+**	[40]
2013	Croatia	**+**	Merdić, pers. comm.
2013	France	**+**	Schaffner, pers. comm.

*No original literature identifiable.

## Review

### Systematics of the Japonicus Group

*Aedes japonicus* occurs with four morphologically very similar subspecies: *Ae. j. japonicus* (Theobald, 1901), *Ae. j. shintienensis* Tsai & Lien, 1950, *Ae. j. amamiensis* Tanaka, Mizusawa & Saugstad, 1979, and *Ae. j. yaeyamensis* Tanaka, Mizusawa & Saugstad, 1979. These varieties may be differentiated by the presence/absence and the particular design of a sub-basal dark band on their hind femora. Secondary characteristics are the colour, alignment and shape of the scales on their posterior pronotal lobes, on their subspiracular areas, on their costae and on their fourth hind tarsomeres [41]. The four subspecies are genetically distinct and form a monophyletic group together with *Aedes koreicus*[42].

### Distribution and spread

The *Ae. japonicus* subspecies have characteristic distribution areas in Asia. *Aedes j. japonicus* is most wide-spread and has been recorded from Palaearctic Japan (Hokkaido, Honshu, Shikoku, Kyushu, Yakushima, Tsushima), the Ryukyu Archipelago, Korea (including both the Korean peninsula and Cheju Island), southern China including Hong Kong, Taiwan and southeastern Siberia (Primorskiy Kray). *Aedes j. shintienensis* is more restricted to the Oriental region, including Taiwan and Korea, while *Ae. j. amamiensis* occurs on Amami Guntô and *Ae. j. yaeyamensis* on Yaeyama Guntô, Okinawa Guntô and associated islands of the Ryukyu Archipelago [41,43,44].

For reasons not quite understood, only *Ae. j. japonicus* has become invasive. In principal, it is supposed to use the same transportation mechanisms as *Ae. albopictus*, primarily the second-hand tyre trade for intercontinental/long-distance overseas movement of the desiccation-resistant eggs [45] and land-based trade and vehicle transport for the dispersal of eggs, larvae and adults on the ground [6,46]. Of course, land-based transport is also possible through displacement of tyres, and it is hypothesized that initial introductions into Ohio and Illinois could be attributed to interstate used tyre commerce [7,27]. It is assumed, however, that a major mode of spread across the USA might be connected to land-based vehicle transport and transit of adult mosquitoes via the Standardbred horse trade [6,47]. Furthermore, Gray et al. [17] and Bevins [48], who found *Ae. j. japonicus* larvae in rock pools associated with all major rivers in the Appalachians and Blue Ridge Mountains of the USA, discuss the possibility of active expansion along river corridors.

For *Ae. albopictus*, introduction of larvae through the lucky bamboo (*Dracaena* spec.) and machinery water and of adults on airplanes has been identified in addition to the used tyre pathway [49,50]. Similarly, *Ae. j. japonicus* was found in used machinery and water tankers arriving in New Zealand [51].

*Aedes j. japonicus* was first detected outside its native distribution range in New Zealand in 1993 [3]. Up to 2003, eight additional introductions were reported but all of them remained interceptions [51]. To date, no establishment of the species in New Zealand has become public.

**Figure 1:**
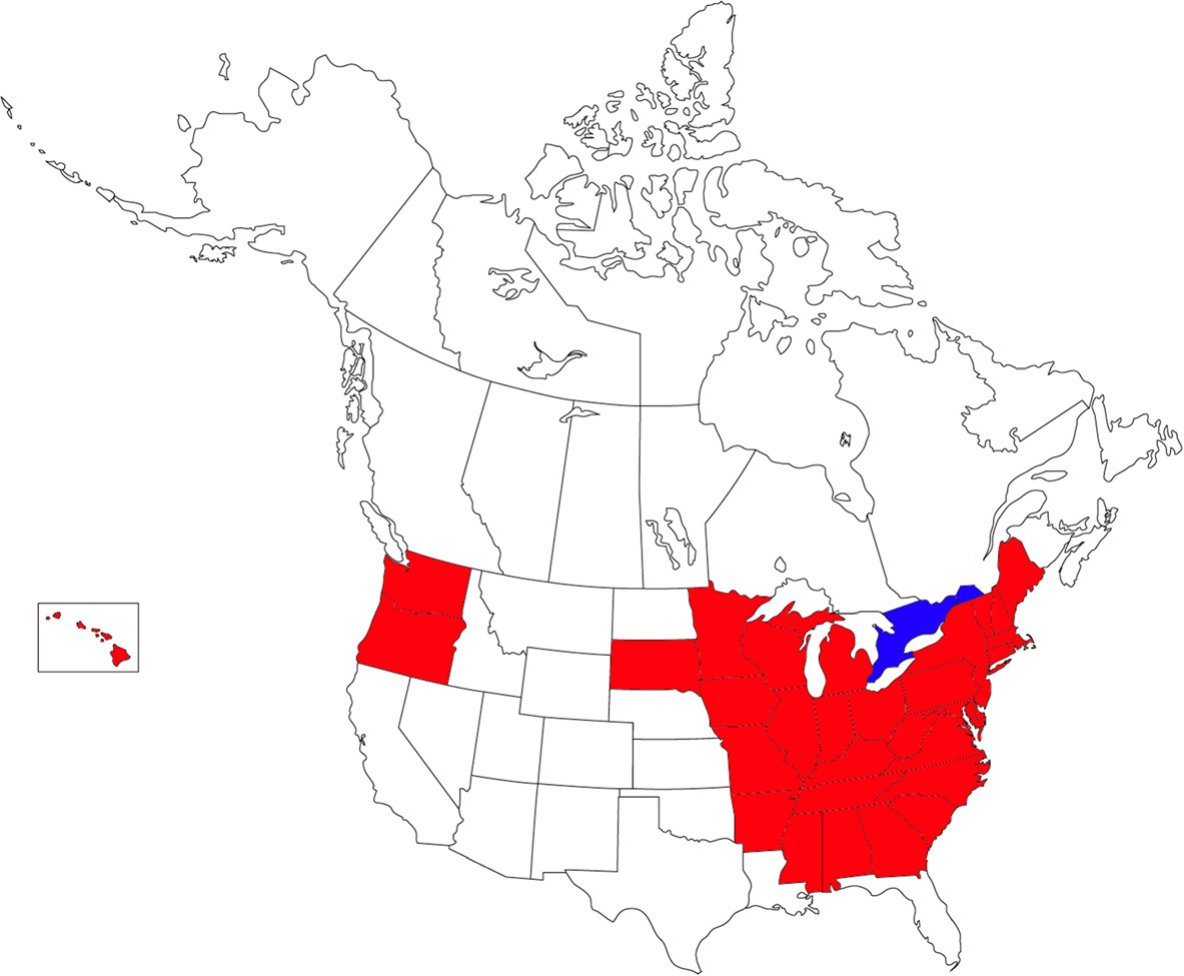
**US states, including Hawaii (box), and Canadian provinces where *Ae. j. japonicus *** **was reported up to 2011 (red and blue colours, resp.) (US states are completely coloured in cases of mosquito detection, Ontario and Quebec provinces in Canada, due to their enormous size, only in those parts where *Ae. j. japonicus *** **was detected).**

In the US, three states notified collections of *Ae. j. japonicus* in 1998, New Jersey, New York and Connecticut [4,5]. Retrospectively, it is not clear when introduction had taken place but it is assumed that it must have been after 1992 since no *Ae. j. japonicus* specimens had been encountered during a previous intense monitoring programme for *Ae. albopictus*[52]. By 2011, *Ae. j. japonicus* was demonstrated in 33 US states including Hawaii (Figure 1, Table 1).

Early phylogenetic investigations by Fonseca et al. [6] on mosquitoes from the eastern US states suggest that there had been at least two independent introductions, since samples from New York, New Jersey and Connecticut represented genetic signatures significantly different from samples from Pennsylvania and Maryland. A more detailed study on mosquitoes collected up to 2005 provided evidence for continuous merging of the initial populations, thus producing increased genetic diversity [53].

While the spread of *Ae. j. japonicus* continued on the eastern US coast and gradually included more central states, establishment of the species was reported from Washington State on the western coast as early as in 2001 [15]. It remained unclear whether this was a result from movement of contaminated products (e.g. tyres) from the eastern states or from another intercontinental introduction event. In 2006, Oregon was the second western state to report occurrence of *Ae. j. japonicus*[28].

In 2001, the northeastern distribution area expanded towards Canada (Figure 1), and the rock pool mosquito was subsequently demonstrated in southern Quebec and southern Ontario [34,35].

In Europe, *Aedes j. japonicus* was first detected in 2000 in the form of two larvae in a storage yard of recycled tyres in a town in northwestern France [36]. According to Schaffner et al. [38], this introduction could be eliminated. In 2002, a used tyre-trading company in Central Belgium was found infested by *Ae. j. japonicus*, and in 2008 the species was discovered on the premises of another Belgian company involved in the second-hand tyre business in the same area [37]. From 2003 onwards, the bush mosquito was regularly collected in Belgium suggesting the establishment of a local population. Interestingly, a spatial spread beyond the companies’ properties was observed only exceptionally although natural breeding sites were accepted in addition to discarded tyres [37]. Control efforts were only initiated in 2012 and continued in 2013, but final eradication could not be confirmed so far (Schaffner, pers. comm.).

Schaffner et al. [38] reported the presence of another European distribution area of *Ae. j. japonicus* in 2008 from northern Switzerland, spreading cross-border into Germany. Prompted by this finding, a monitoring programme carried out in southern Germany demonstrated a wide-spread population in 2009 with further expansion up to 2012 [54-56], (Becker, pers. comm.). Additional populations were found in 2012 in western Germany [57] and in 2013 in northern Germany [58] after individual mosquitoes were submitted to the authors for identification by private persons. Since the West German area was considerably larger and more densely populated than the North German one, it was assumed to be older. Most of the positive sites in northern Germany were located close to a motorway connecting the two distribution areas, suggesting that the North German population was probably an offshoot of the West German one [58]. Apparently, the Swiss/South German population expands westwards as *Ae. j. japonicus* specimens were detected in southern Alsace in 2013 (Schaffner, pers. comm.).

In 2011, immature bush mosquito stages were also collected in the border region of Austria (Styria) and Slovenia [39]. During a monitoring in Slovenia carried out in 2013, the whole north-eastern half of Slovenia was found colonized by *Ae. j. japonicus*[59], with frontier crossing to Croatia where several specimens were detected in 2013 close to the Slovenian border (Merdić, pers. comm.).

A single *Ae. j. japonicus* specimen was trapped in the Central Netherlands during routine monitoring in 2012, prompting a more thorough surveillance in the affected municipality with a closer trap grid in 2013. When several females were found, a site inspection produced numerous breeding sites containing preimaginal stages [40].

**Figure 2:**
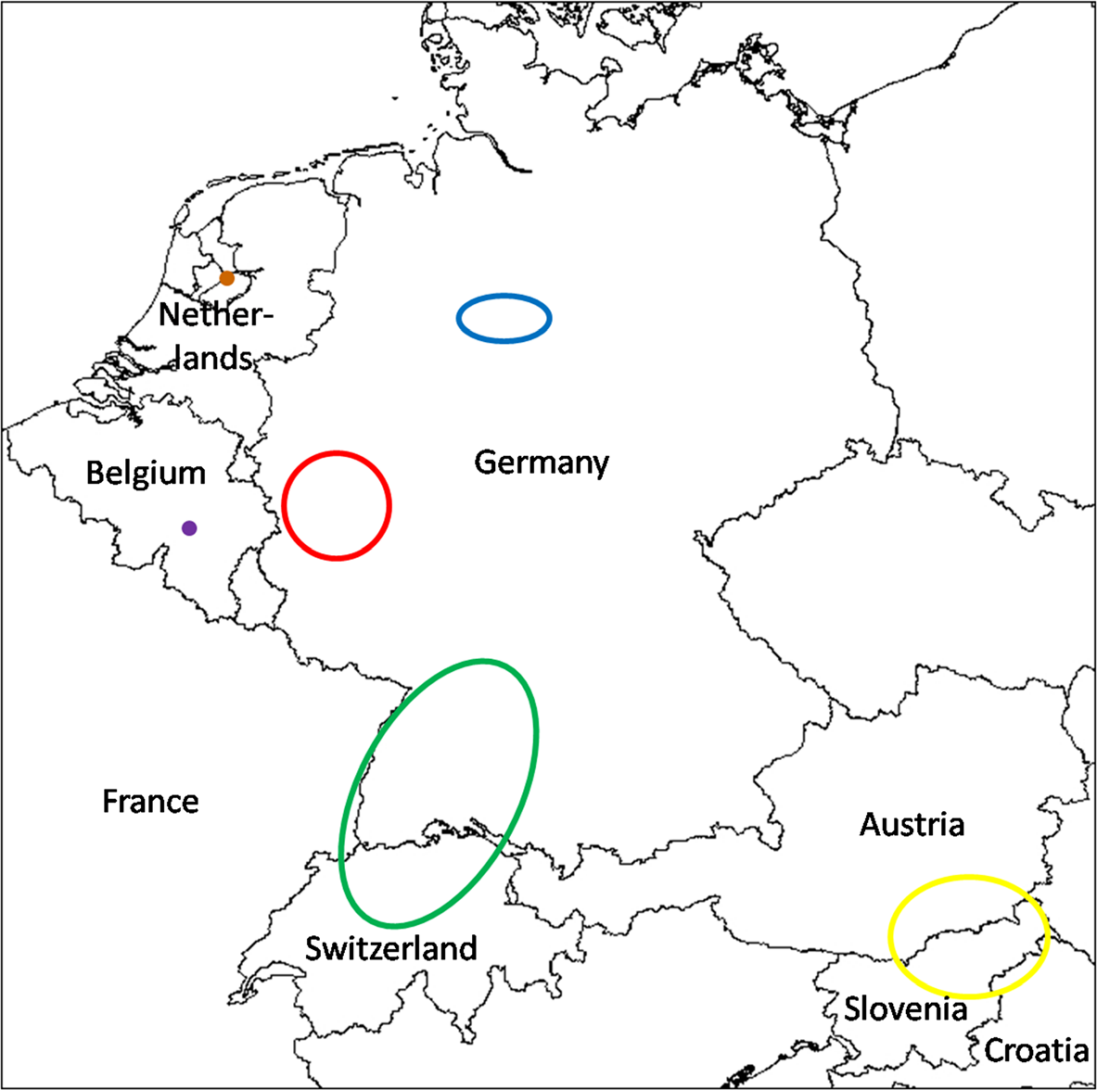
**European distribution areas of *Ae. j. japonicus *** **as of late 2013.**

In summary, it appears that six spatially separated colonization areas exist in Europe: Belgium (being treated), northern Switzerland/France/southern Germany, western Germany, northern Germany, Austria/Slovenia/Croatia and the Central Netherlands (Figure 2). Except for Belgium, it is not known where the mosquitoes came from and how they were imported [e.g. [38,57]. It is also not clear whether the various European populations are related to each other and whether they go back to different introduction events. Population genetic analyses using microsatellite signatures are underway to shed light on their relationships and geographic origins.

### Biology

*Aedes j. japonicus* is a mosquito species adapted to temperate climates, capable of withstanding cold and snowy winters as occuring in its endemic home range in northern Japan. According to Kamimura [60], overwintering takes place as eggs in northeastern Japan and as larvae in southwestern Japan. In the southern Appalachians, USA, larvae were found at altitudes of up to 1,500 m where winter temperatures can reach −18°C [48].

The life-cycle is multivoltine both in Japan [61] and in the eastern US [52]. During a five-year period in southern New Hampshire, Burger & Davis [9] observed hatching of larvae at the end of March, as soon as breeding sites lost their ice cover, at water temperatures of 4.0-4.5°C. Larvae were present until freezing produced a new ice cover in late October or early November. In laboratory studies larvae were demonstrated to hatch at 5°C water temperature and to be able to survive extended periods at low temperatures (e.g. 141 days at 10°C [8]). These observations suggest that *Ae. j. japonicus* can indeed overwinter in the larval stage as communicated by Kamimura [60] for southern Japan.

The activity period of adults may last from May to October [62,63] or, at mild temperatures, even to early December [64]. At 22°C under laboratory conditions, preimaginal development, i.e. the period from larval hatching to adult emergence, takes two to four weeks [8]. Females exhibit a crepuscular/diurnal biting habit [65,66] and are assumed to have a relatively short flight range [6].

*Aedes j. japonicus* has a preference for forested and bushy areas, be they in agricultural, rural, suburban or urban settings [41,52]. Being essentially a rock-hole breeder in its native range [41], streamside rock pools are among numerous other natural and movable or stationary artificial containers colonized elsewhere. Larvae can be found in tree-holes, leaf axils, tree stumps, rain water pools, ditches, subterranean catch basins, used tyre casings, stone vessels, drainage pipes, drinking fountains, tin cans, bath tubs, bird baths, roof gutters, flower vases, water dishes for pot plants, plastic cartons, vinyl tarpaulins, street gutters, rain water barrels, buckets, pans, etc. [16,38,48,52,62,67-72]. Strikingly, the breeding habitats often contain decaying leaves, twigs, other decomposing organic matter and algae [41,52,62]. Larval collections in extremely dirty artificial containers, such as used ashtrays, indicate a high tolerance to environmental pollutants [35].

Breeding sites are selected in both sun-lit and shaded environments [41,52], suggesting that the larvae and pupae can tolerate a wide range of water temperatures [71]. However, confirming the mosquito’s distribution boundaries in Japan [41], the frequent lack of *Ae. j. japonicus* in warm water pools fully exposed to the sun in some areas led Andreadis & Wolfe [73] to suggest that a temperature barrier may exist for this species preventing the colonization of regions with relatively high summer temperatures. This may explain the present, more northerly distribution areas in Europe and North America. In the southern US state Alabama, for example, a single *Ae. j. japonicus* female was trapped in the northeasternmost corner in 2005, whereas no specimens at all were encountered in any other county during a state-wide survey of tyre-breeding mosquitoes [74]. Also in Europe, establishment has as yet been demonstrated only in the central countries where a temperate climate prevails, but not in southern subtropical regions.

In Japan, *Ae. j. japonicus* females are said to be reluctant to bite humans [41,62], whereas they readily feed on chicken and mice in the laboratory, but not on reptiles or amphibians [75]. Williges et al. [76] feed their laboratory colony on guinea pigs, and Takashima & Rosen [77], Sardelis & Turell [11] and Sardelis et al. [78-80] used chickens, hamsters and mice, respectively, as blood hosts for transmission studies. In contrast to extensive biting study observations in the field by LaCasse & Yamaguti [62] where *Ae. j. japonicus* was never encountered, Iriarte et al. [61] reported *Ae. j. japonicus* to be abundant near human dwellings in urban areas, and Kamimura [81] and Andreadis et al. [52] actually observed attraction to, and even attacks on, humans in the field, indicating a feeding purpose. Knight [67] also argues that the species will readily bite humans entering their habitats. While analyzing blood meals from wild-caught *Ae. j. japonicus*, Apperson et al. [82] found that all tested samples were of mammalian but none of human origin. Molaei et al. [83], however, demonstrated acquisition of blood from human hosts in a third of examined blood-engorged females. In summary, there is evidence that the bush mosquito feeds on both avian and mammalian, including human, hosts. Vector competence provided, this indiscriminant behaviour makes it a potential bridge vector of zoonotic viruses such as West Nile virus (WNV).

Once established under adequate climatic conditions, *Aedes j. japonicus* may quickly propagate. In some newly infested areas, the invader was detected quite early, either by chance or as a consequence of routine mosquito surveillance, when population densities were still low and distribution was limited. It has commonly been observed that detection frequency, together with breeding site numbers and spatial distribution, will usually increase significantly within one to three years after the initial phase of colonization, resulting from considerable population growth [9,20,22,27,35]. However, the initial phase of colonization is missed in some areas, and the mosquito’s presence is only recognized with some delay, probably a few years, when high population numbers and a wide-spread distribution have already been reached [e.g. [5,11,52].

Along with the observed adaptive capacity and successful proliferation of *Ae. j. japonicus* in many areas of North America and Europe comes evidence that it represses the population densities of other culicids [9,57,73,84]. Such a displacement of indigenous mosquito species would of course directly affect the biodiversity and may also have direct or indirect effects on the epidemiology of mosquito-borne diseases [48,85]. While competitive advantage could not be proven so far or remains anecdotal in Europe [38,57], there is evidence from the US that *Ae. j. japonicus* larvae outcompete larvae of other species. A decline in relative abundances as compared to *Ae. j. japonicus* has been reported for *Aedes atropalpus, Aedes triseriatus, Culex restuans* and even *Culex pipiens*[22,72,73,84-86]. Handicaps of *Ae. atropalpus*, for example, are hypothesized to be autogeny and a longer larval development [85]. *Aedes triseriatus* is inferior in sunlit peridomestic locations [72] and *Cx. pipiens* when decaying leaves and algae are the only food sources [86]. Similar negative effects with weak support for *Ae. j. japonicus* over *Ae. triseriatus* with regard to development time were also observed by Alto [87] when these two species were co-bred under artificial conditions. These observations contrast somewhat with studies which indicate that *Cx. pipiens* maintained similar larval populations in tyres before and after the introduction of *Ae. j. japonicus* in Connecticut [73] and with studies of larval competition between *Ae. j. japonicus* and *Cx. pipiens* reared on laboratory diets which indicate that the two species are equivalent competitors [88].

Being possibly superior in competition to a number of native culicid species, *Ae. j. japonicus* larvae have been shown in laboratory experiments to be inferior to the Asian tiger mosquito *Ae. albopictus*[89], another invasive container-breeding culicid species which had started its worldwide spread long before the Asian bush mosquito [90-92]. This would be unfortunate if transferable to natural conditions since *Ae. albopictus* is a highly efficient vector of dirofilarial nematodes and more than 20 arboviruses including many of public health relevance [93]. It is therefore thought to pose a considerably greater threat to invaded countries than *Ae. j. japonicus*. Field observations made by Bartlett-Healy et al. [94] do not necessarily support the laboratory results of Armistead et al. [89]. They rather suggest that species abundance depends on urban, suburban or rural surroundings, with *Ae. albopictus* being more abundant in urban and suburban settings, and *Ae. j. japonicus* being more abundant in rural settings when both are sympatric.

There are most likely numerous factors to be considered simultaneously when larval competition is to be determined, such as food resource availability and quality, water quality, shade and insolation, and temperature [94]. Thus, the competition between *Ae. j. japonicus* and *Aedes epactius*, another US resident mosquito species, appears to be clearly dependent on season-associated temperature [32]. Early in the year, when temperatures are cooler, *Ae. j. japonicus* is the dominating species in rock hole habitats while later in the year, during the hotter months, *Ae. epactius* is more frequent.

### Vector potential

*Aedes j. japonicus* is not considered an important vector in its native Asian distribution area, and evidence for a major role in field transmission of disease agents is generally absent. Some data suggesting a vector potential for several viruses of medical and veterinary relevance, however, exist both from the field and from laboratory infection and transmission studies (Table 2). While Russian and Japanese researchers were interested in the susceptibility of *Ae. j. japonicus* to Japanese encephalitis virus (JEV), which is endemic in many East Asian countries where the mosquito occurs, long before the discovery of the mosquito becoming invasive, western colleagues have mainly addressed the question of vector competence only after the emergence of the bush mosquito on other continents (Table 2).

**Table 2:** **Demonstrated role of *Ae. j. japonicus *** **in pathogen transmission**

	**Field transmission**	**Field infection**	**Laboratory transmission**	**Laboratory infection**	**Reference**
**West Nile virus**	?	+	+	+	[11,95-97]
**Japanese encephalitis virus**	?	+	+	+	[77,98-100]
**St. Louis encephalitis virus**	?		+	+	[80]
**Eastern equine encephalitis virus**	?		+	+	[78]
**La Crosse virus**	?		+	+	[79]
**Rift Valley fever virus**	?		+	+	[101]
**Chikungunya virus**	?			+	[102]
**Dengue virus**	?			+	[102]
**Getah virus**	?			+	[103]

Thus, Mitamura et al. [98] reported experimental JEV infection and subsequent transmission by *Ae. j. japonicus* in Japan decades ago, whereas the mosquitoes became infected but did not transmit the virus in a Russian study by Petrischeva & Shubladse [99]. Specimens positive for JEV were also found in the field in Russia [100]. Takashima & Rosen [77] have later shown the capability of *Ae. j. japonicus* of transmitting the virus both horizontally and vertically in the laboratory. In the field, its vector role for JEV may be obscured by the principal vector, *Culex tritaeniorhynchus*, which breeds in the countless Asian rice fields. *Aedes j. japonicus* probably may only gain some significance as a vector in areas devoid of rice fields and *Cx. tritaeniorhynchus*[104]. JEV causes serious encephalitides in humans and horses and abortion in pigs [105].

Turell et al. [95,96] and Sardelis & Turell [11] have shown laboratory transmission of WNV by feeding *Ae. j. japonicus* on viremic chickens and re-feeding them on naïve chickens after a period of two weeks. WNV affects birds, equines and humans, where infections may go asymptomatically or cause encephalitis, meningitis, flaccid paralysis and even death. Epidemics have occurred in Europe, Africa, North and Central America, the Middle East and parts of Asia [106]. Major WNV vectors are *Culex* species but the virus was found in numerous field-collected pools of *Ae. j. japonicus* from at least nine different US states between 2000 and 2009 [83,97].

Sardelis et al. [78-80] also described experimental transmission of eastern equine encephalitis virus (EEEV), La Crosse virus (LACV) and St. Louis encephalitis virus (SLEV) by *Ae. j. japonicus* fed on viremic chickens or hamsters first and on naïve chickens or suckling mice one to three weeks later, respectively. Most human infections with all three viruses remain subclinical or inapparent, while in some cases they may involve the central nervous system and cause severe encephalitides and death. LACV and EEEV are restricted to North America where the principal mosquito vectors are *Culiseta melanura* and *Ae. triseriatus*, respectively [107,108]. SLEV is distributed throughout the Americas and is transmitted by various *Culex* species [109].

Quite recently, Turell et al. [101] also demonstrated efficient laboratory transmission of Rift Valley fever virus (RVFV) by *Ae. j. japonicus*. More than 90% of all mosquitoes fed on viremic hamsters had disseminated infections from seven days post infection onwards, and virus transmission to susceptible hamsters readily occurred. RVFV virus is highly pathogenic to humans and ruminants [110,111] and raises great concern that it might be imported into Europe and North America [112,113]. It has long been endemic to sub-Saharan Africa but epidemics have increasingly been occurring in North Africa and the Middle East [112]. Notably, RVFV is also transmitted transovarially by some mosquito species. Infected females pass the virus over to the next generation via the eggs resulting in the fact that the virus can persist in the mosquito population over extended periods of time without involvement of a vertebrate host [114].

Susceptibility to infection, but not virus transmission, has been shown for chikungunya and dengue viruses by Schaffner et al. [102], by artificial feeding of *Ae. j. japonicus* on viremic blood through chicken skin and saliva titration 14 days later. Occurrence of the viral particles in the saliva indicated vector competence. Both viruses have been proven to be transmitted by synanthropic *Aedes* species, such as *Ae. albopictus* and *Aedes aegypti*, and have recently caused large unexpected outbreaks in northern Italy (chikungunya [115]) and on the Portuguese island of Madeira (dengue [116]), respectively. Chikungunya may be associated with long-lasting and painful polyarthralgia [117] while dengue may present with a hemorrhagic fever or a shock syndrome and be fatal [118].

Getah virus occurs in southeastern Asia and Australia, is transmitted by various *Aedes* and *Culex* species, and has been described as causing disease in horses, characterized by pyrexia, urticaria, rash and oedema of the hind legs [119]. *Aedes j. japonicus* showed susceptible to the virus when fed on viremic blood through pig skin membrane as demonstrated by titration of whole mosquito homogenates 21 days after blood ingestion [103].

## Conclusion

Although being capable of transmitting pathogenic viruses in the laboratory, *Aedes j. japonicus* has not been confirmed to be a vector of disease agents in the field. Whether there are intrinsic factors generally preventing the species from becoming a conspicuous and efficient vector or whether the non-appearance to date of *Ae. j. japonicus* as a vector is by chance, due to the mosquito and dangerous pathogens not being endemic in the same regions, remains to be elucidated.

*Aedes j. japonicus* seems to be highly adaptive and to have competitive advantages over some mosquito species indigenous to the invaded regions. In the long-term, this may not only have direct effects on the biodiversity by a change of the mosquito fauna but may also have indirect impacts on the epidemiology of mosquito-borne diseases should they occur in the infested regions.

In many cases, in particular in Europe, the origin, the port(s) of entry and the modes of continental transportation of *Ae. j. japonicus* are unknown. More ecological, population genetic and vector competence studies are fundamental to a better understanding of the establishment and spread of *Ae. j. japonicus* and an assessment of its impact on public health. In this context, biological and ecological data at the natural range of distribution in Asia and overseas regions where the species has become established (North America and Europe) could be used to develop predictive distribution maps for countries where it has not been already introduced.

Given the wide distribution of established populations, both in North America and Europe, it is wishful thinking that *Ae. j. japonicus* can again be eradicated. Instead, it is imperative to accept it as having become a part of the indigenous mosquito fauna and to try to keep its population densities at a possible minimum by educating and training community workers, personnel involved in gardening and landscaping etc., as well as the general public as to how to avoid producing potential breeding sites.

## Abbreviations

CDC: Centers for Disease Control and Prevention; EEEC: Eastern equine encephalitis virus; JEV: Japanese encephalitis virus; LACV: La Crosse virus; RVFV: Rift Valley fever virus; SLEV: St. Louis encephalitis virus; WNV: West Nile virus.

## Competing interests

The authors declare that they have no competing interests.

## Authors’ contributions

Both authors contributed equally to the literature research and writing of the manuscript. Both authors read and approved the final manuscript.
